# Nonlinear oscillations of a lumped system with series spring, piezoelectric device, and feedback controller

**DOI:** 10.1038/s41598-025-97173-2

**Published:** 2025-04-26

**Authors:** M. K. Abohamer, T. S. Amer, A. A. Galal, Mona A. Darweesh, A. Arab, Taher A. Bahnasy

**Affiliations:** 1 Department of Engineering Physics and Mathematics, Faculty of Engineering, Tanta University, Tanta, 31734 Egypt; 2 Mathematics Department, Faculty of Science, Tanta University, Tanta, 31527 Egypt; 3 Chemical and Petrochemical Engineering Department, Faculty of Engineering, Tanta University, Tanta, 31734 Egypt

**Keywords:** Nonlinear dynamics, Lumped system, Negative controller, Asymptotic analysis, Perturbation analysis, Resonance, Piezoelectric device, Harvesting, Modulation equations, Stability, Bifurcation analysis, Poincaré maps, Mechanical engineering, Applied mathematics

## Abstract

This paper examines the behavior of a mechanical system with a lumped- mass comprising two nonlinear springs arranged in series and combined with a piezoelectric device. External harmonic excitations, as well as linear and nonlinear damping, are considered. The main system employs a negative velocity feedback (NVF) controller to reduce undesired effects vibrations, particularly under resonance conditions, thereby enhancing the system’s efficiency. The system is described by differential and algebraic equations, forming a dynamic model governed by differential-algebraic equations (DAE). A nearly analytical technique is further applied to resolve the initial value problem of the DAE. Applying the Lagrange’s equations (LE), the regulating equations of motion (EOM) are derived. The approximate solutions (AS) to third-order are obtained subsequently in the framework of the multiple-scales method (MSM). The AS’s accuracy is confirmed by comparing it to the numerical solution (NS) obtained via Runge–Kutta fourth-order algorithms (RK- 4). Examining the resonance cases, along with the criteria of solvability, leads to the derivation of the modulation equations (ME). Graphical representations of the solutions’ time histories and frequency response curves are presented using Wolfram Mathematica 9 and MATLAB- 23 software, providing a thorough visualization of the results. In addition, bifurcation diagrams and Poincaré maps (PMs) are graphed to illustrate the different behavioral modes of the system. Conversely, piezoelectric transducers are linked to the dynamic model to transform vibrational motion into electrical energy. This technology represents one of the many energy harvesting (EH) solutions widely utilized across commercial, aerospace, industrial, medical sectors, and automotive. A graphical analysis illustrating the time courses of solutions with and without control is presented. Additionally, resonance frequency curves are plotted to assess stability/instability and evaluate the solutions at steady-state.

## Introduction

Mechanical systems with massless springs arranged in parallel or series have been extensively studied in applied and theoretical mechanics. These systems are widely used in disciplines such as mechanical engineering, construction, mechatronics, and, as of late, micromechanical systems. The configuration of spring connections, including their spatial orientation, can result in complex dynamic behavior, particularly when the springs exhibit nonlinear properties. Such systems often display intriguing and sometimes unexpected behaviors, especially in relation to resonant states. Furthermore, the significance of vibration control has considerably increased in recent decades, becoming a pivotal development in lightweight and low-damped structures designed to reduce vibrations. Through the prevention of potential damage or catastrophic failures, this method supports the extended lifespan of these systems. Numerous researchers and scientists have focused their efforts on addressing this issue, which impacts equipment, industry, and economic efficiency.

Modeling many real-world systems necessitates the use of a rigid body approximation involving the connection of springs and dampers in different configurations^1,2^. studied a car suspension with parallel and series spring systems. The authors demonstrated that this connection significantly affects how vibrations from rough roads are transmitted to the body. In^3^, the authors analyzed a supported single DOF oscillator by two springs: one with linear properties and the other with nonlinear characteristics. For the examined system, they presented two mathematical models. The first approach utilizes DAE, which has been solved through numerical methods. Unlike the first, the second model, formulated from a single differential equation with relative displacement variables, offers an approximate analytical solution. By comparing the outcomes from both methods, they identified the parameter values that characterize the elastic properties of the springs, revealing a significant level of agreement.

The proposal in^4^introduces a feedback control system to regulate displacement and velocity, addressing time delay, in order to enhance the stability of a giant magnetostrictive actuator system. Moreover, this approach handles the nonlinear dynamic properties, which encompass the primary frequency response, chaotic motion, and limiting cycle amplitude. Giant magnetostrictive materials, a novel category of substances, have gained extensive use in applications such as EH, aviation, electronic controls for gasoline injection, and electro-hydraulic servo valves, among others^5–10^. In^11^, a nonlinear dynamic differential equation related to a cable-stayed beam under the influence of time-delayed feedback was formulated by the authors. Their analysis demonstrated that velocity feedback control was less effective than both displacement and acceleration feedback control. Moreover, in^12^, a model for torsional vibrations in an electromechanical connection transmission system was established. The study noted that increasing the feedback gain led to a decreased instability region, facilitating a shift from chaotic motion to periodic motion.

A groundbreaking analysis has been conducted, exploring how a quarter-vehicle transportation system responds to shifts in natural frequency. The multiple-scale homotopy method was employed to obtain a mathematical solution for the equations of the controlled system. As a renewable energy source, EH is especially noteworthy for its capability to harness and convert waste ambient energy into a practical form^13–15^. In^16^, the authors examined a dynamical system of three degrees of freedom. The EOM were derived using LE, and the AS was assessed through MSM. The comparison of AS with numerical results validated the reliability of the former. The evaluation of resonance conditions and solvability criteria led to the determination of ME. Various forms of motion within the system, along with PMs, were characterized through bifurcation plots and Lyapunov exponent spectrums.

In^17^, a dynamic model was proposed for the shearer’s permanent magnet small drive braking system, incorporating a multi-excitation force mechanism. The study addressed and resolved the complexities associated with this mechanism. Furthermore, the adverse effects of resonance incidents were analyzed through the application of a cubic velocity feedback (CVF) controller and a NVF controller. The interaction between the NVF and CVF controllers was elucidated, revealing that approximately 99.95% of the control impact can be attributed to the NVF controller, while around 96.7% is linked to the CVF controller. The NVF controller proved to deliver superior performance under the influence of both parametric and external excitation forces in the system. In^18^the authors introduce Nonlinear enhanced positive position feedback control for a cantilever beam system with an intermediate lumped mass. Control of 1/3 order subharmonic resonance in a mass-damper-spring system using cubic-position and NVF is investigated in^19^.

In^20^, a mathematical model was explored regarding a spinning beam operating at various speeds. The nonlinear system of differential equations was analyzed under the influence of the MSM, focusing on the approximate solutions for the system’s behavior during resonance conditions. The investigation included the implementation of a proportional-derivative controller to facilitate delayed control of displacement and velocity. In^21^MSM analysis is studied for predicting quasiperiodic oscillations in a thin-walled beam under simultaneous resonance. Reference^22^presented a study on a coupled pitch-roll ship model utilizing CVF control in response to parametric excitation. In references^23–32^, the EOM for the system were derived using LE and subsequently solved with the MSM. The primary external resonance scenario under consideration led to the formulation of modulation equations and conditions for solvability. The analysis incorporated specific variable values to generate time histories and resonance curves. Additionally, the authors of reference^27,28^ utilized EH devices to convert vibrations into electrical energy.

This work combines a piezoelectric EH device with a lumped system that includes a series spring with the goal of transforming vibrational motion into electrical energy. The NVF controller is designed to minimize the arising harmful vibrations during resonant conditions. Using LE, we derive the primary EOM, while the piezoelectric circuit mechanism supplies the corresponding equations. The MSM is utilized to obtain the AS up to the third-order. Numerical simulations are performed using the RK- 4 method to compute the NS, and the graphical results are compared with the AS to evaluate the efficacy of the MSM. After eliminating secular terms, we establish the solvability criteria and explore all resonance scenarios. By considering the NS of the ME, one demonstrates the time evolution of the modified amplitudes and phases. Moreover, the stability/instability areas around the frequency response curves are analyzed. Through bifurcation diagrams and PMs, the system’s behavior is thoroughly examined. The achieved results are noteworthy, as they analyze the vibrational dynamics of a lumped system with the purpose of harvesting usable electrical energy for practical applications using a piezoelectric device.

## Analytical representations of the oscillator

**Fig. 1:**
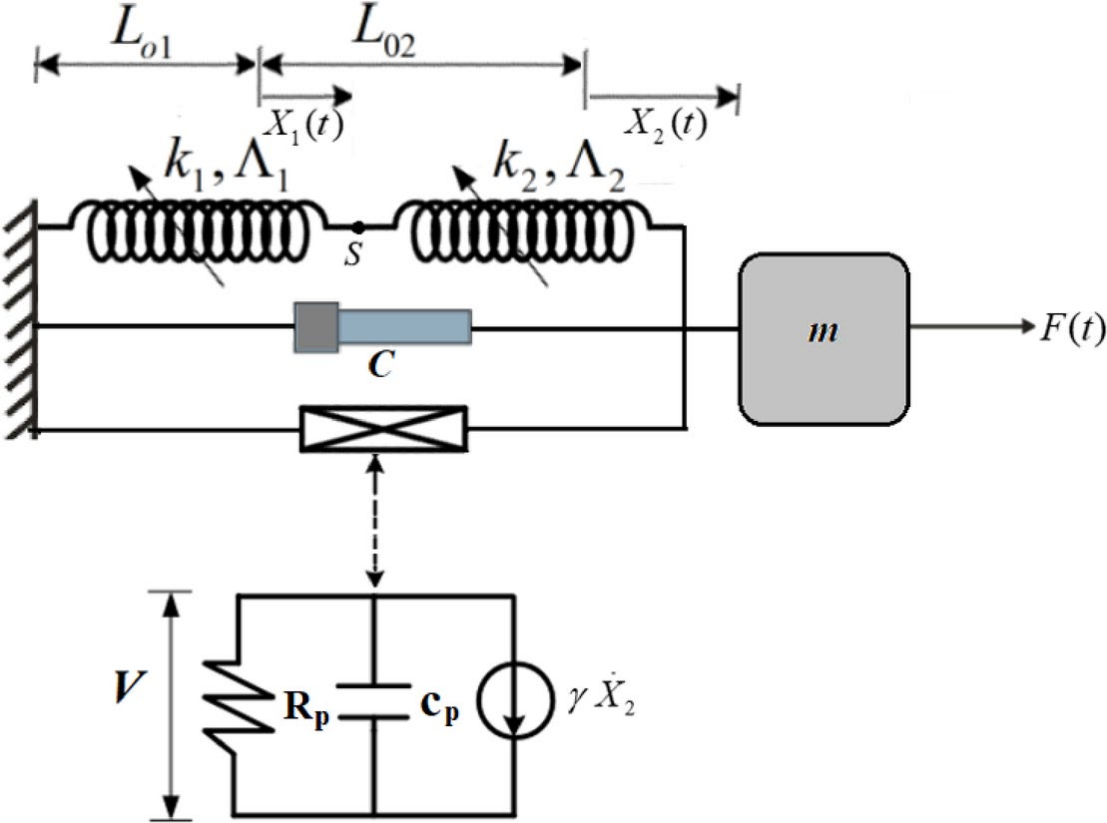
Graphical representation of the dynamical system.

**Fig. 2:**
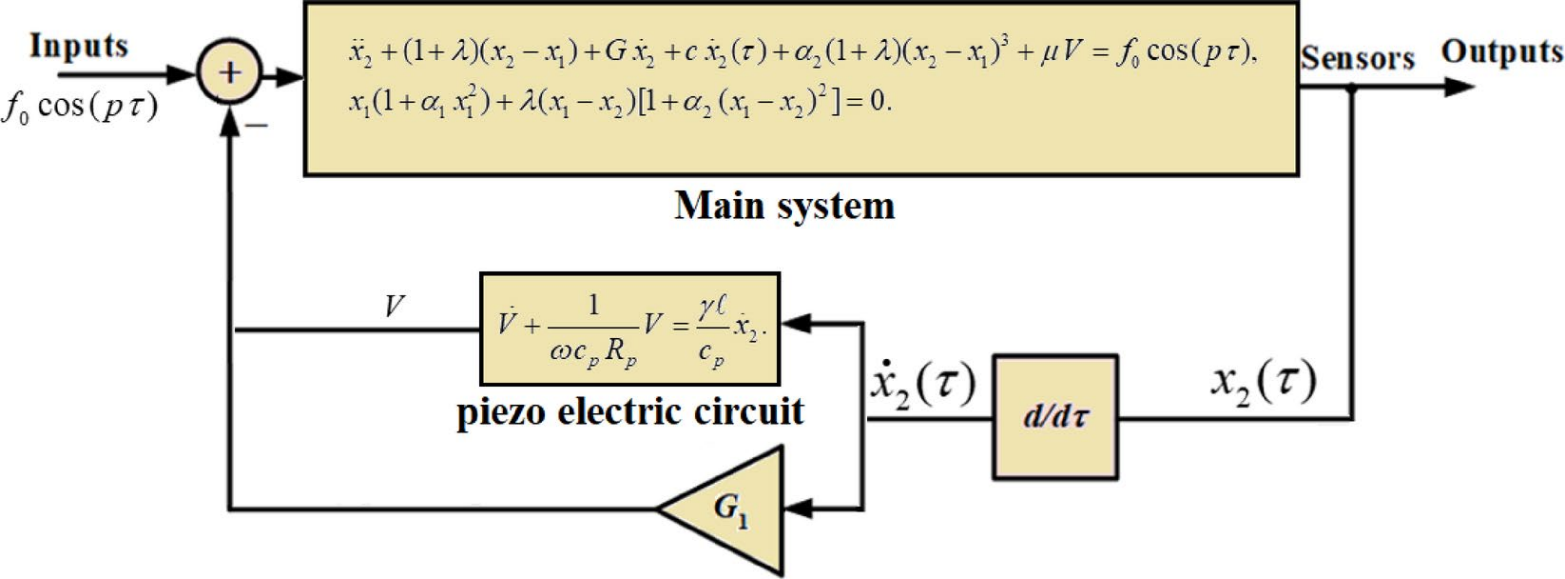
Demonstrates the NVF controller for the dynamic model.

A rigid body with mass m is constrained to move in the horizontal direction. It is linked to a fixed wall through two springs arranged in series, along with a viscous damper characterized by a damping coefficient C. Additionally, it is subjected to an external force represented by the harmonically varying function $F(t)={F_0}\cos \,(\Omega \,t)$, alongside a piezoelectric circuit with a capacitance and a resistive load ${c_p}$ and ${R_p}$, respectively. The studied system is illustrated in Fig. 1. To mitigate the rising fluctuation amplitudes during resonance, an NVF controller with a specified gain ${G_1}$ is employed, as depicted in Fig. 2. The massless point S, positioned where the springs are joined together, is given by ${L_{o1}}+{X_1}(t),$ while the position of the body is defined as ${L_{o1}}+{L_{o2}}+{X_2}(t),$ where the symbols ${L_{o1}}$ and ${L_{o2}}$ indicate the springs’ nominal lengths.

The kinetic energy T of the mentioned system can be represented in a specific form.

1 $$T=\frac{1}{2}{\dot {X}_2}.$$

The springs exhibit nonlinear cubic characteristics, leading to the potential energy ${V_E}$ being represented accordingly.

2 $${V_E}={k_1}(\frac{1}{2}X_{1}^{2}+\frac{1}{4}{\Lambda _1}\,X_{1}^{4})+{k_2}[\frac{1}{2}{({X_2} - {X_1})^2}+\frac{1}{4}{\Lambda _2}\,{({X_2} - {X_1})^4}],$$

where ${k_i}\,\,(i=1,2)$ and ${\Lambda _i}$ are the ${i^{th}}$ spring’s stiffness coefficients.

The generalized force is thought to consist of the damping force and the external excitation.

3 $$Q={F_0}\cos \,(\Omega \,t) - C{\dot {X}_2}.$$

The equilibrium equation for point S, which is massless, must be satisfied because the springs are connected serially

4 $${k_1}{X_1}(1+{\Lambda _1}\,X_{1}^{2})+{k_2}({X_1} - {X_2})[1+{\Lambda _2}\,{({X_1} - {X_2})^2}]=0.$$

The LE helps derive the EOM for the system, which is then transformed into a more practical dimensionless format. Similarly, the equilibrium equation is presented in dimensionless notation. The governing equations, incorporating the NVF control, are expressed as follows^33^

5 $${\ddot {x}_2}+(1+\lambda )({x_2} - {x_1})+c\,{\dot {x}_2}+{\alpha _2}(1+\lambda ){({x_2} - {x_1})^3}+\mu \,V={f_0}\,\cos \,(p\,\tau ) - G\,{\dot {x}_2},$$

6 $${x_1}(1+{\alpha _1}\,x_{1}^{2})+\lambda ({x_1} - {x_2})[1+{\alpha _2}\,{({x_1} - {x_2})^2}]=0.$$

Additionally, the mechanism equation^27^ for the circuit of the piezoelectric has the form

7 $$\dot {V}+\frac{1}{{\omega \,{c_p}\,{R_p}}}V=\frac{{\gamma \ell }}{{{c_p}}}{\dot {x}_2},$$

where $\gamma$ is the coupling coefficient between the dynamical system and the harvesting device.

Now, the parameters without dimensions will be examined as follows

8 $$\begin{gathered} \omega =\sqrt {\frac{{{k_e}\,}}{m}} ,\,\,\,\,\,p=\frac{\Omega }{\omega },\,\,\,\,\lambda =\frac{{{k_2}}}{{{k_1}}},\,\,\,\,\tau =\omega \,t,\,\,\,\,\mu =\frac{\gamma }{{m\,\omega \,\ell }}\,\,\,\,{x_i}=\frac{{{X_i}}}{\ell },\,\,\,\, \hfill \\ c=\frac{C}{{\omega \,m}},\,\,\,\,{k_e}=\frac{{{k_1}{k_2}}}{{{k_1}+{k_2}}},\,\,\,\,{f_0}=\frac{{{F_0}}}{{\omega \,m\,\ell }},\,\,\,\,{\alpha _i}={\Lambda _i}{\ell ^2},\, \hfill \\ \end{gathered}$$

where $$\ell ={L_{o1}}+{L_{o2}}$$.

It should be mentioned that the following factors make establishing parameters in dimensionless forms crucial for vibrating dynamical systems:

The differential equations controlling the vibrating system are frequently made simpler by these forms, which facilitates the tractability of analytical and numerical solutions. The system’s response is greatly enhanced in key resonance conditions when dimensionless parameters are utilized. This is crucial when designing systems to either mitigate or benefit from resonance. In addition, dimensionless parameters allow for the scaling of results, enabling the application of model system findings to real-world systems of various sizes or materials. These factors make it simpler to compare various vibrating systems or configurations, which aids in determining the best designs or setups for certain uses.

## The proposed method

In this section, the MSM is used to obtain the AS of the previously provided system of Eqs. (5)-(7). Therefore, we focus our study on the dynamic behavior of this system in a narrow region bounded by the static equilibrium point^34^. Then, the following is an expression for its vibrational amplitudes:

9 $${x_1}(\tau )=\varepsilon \,{\chi _1}(\tau ;\varepsilon ),\,\,\,\,{x_2}(\tau )=\varepsilon \,{\chi _2}(\tau ;\varepsilon ),\,\,\,\,V(\tau )=\varepsilon \,v(\tau ;\varepsilon ),\,\,\,\,$$

in which $0<\varepsilon <<1$ is a small parameter. Considering, the following variables and parameters were as follows

10 $$\begin{gathered} \mu ={\varepsilon ^2}\,\tilde {\mu },\,\,\,\,\,c=\,{\varepsilon ^2}\,\tilde {c},\,\,\,\,\,G=\,{\varepsilon ^2}\,\tilde {G},\,\,\,\,\,{c_p}\,={\varepsilon ^2}{{\tilde {c}}_p},\,\,\,\,\,f=\,{\varepsilon ^2}\,\tilde {f}\,\,\,\,\,\,\,\,\,\,\, \hfill \\ {\alpha _i}={\varepsilon ^2}\,{{\tilde {\alpha }}_i},\,\,\,\,\,{R_p}={{{{\tilde {R}}_p}} \mathord{\left/ {\vphantom {{{{\tilde {R}}_p}} {{\varepsilon ^2}}}} \right. \kern-0pt} {{\varepsilon ^2}}}. \hfill \\ \end{gathered}$$

According to the MSM, the answers can be expressed as follows^35^:

11 $$\begin{gathered} {\chi _j}=\sum\nolimits_{{k=0}}^{2} {{\varepsilon ^k}{\chi _{jk}}({\tau _0},\,{\tau _1},\,{\tau _2})} +O({\varepsilon ^3}),j=1,2, \hfill \\ v=\sum\nolimits_{{k=0}}^{2} {{\varepsilon ^k}{v_k}({\tau _0},\,{\tau _1},\,{\tau _2})} +O({\varepsilon ^3}). \hfill \\ \end{gathered}$$

The fast scale is represented here by ${\tau _0}$ and while the slower ones are indicated by ${\tau _1}$ and${\tau _2}$. Now, derivatives must be changed $\tau$ in relation to these scales. Therefore, to achieve this goal, the differential operators listed below can be utilized

12 $$\begin{gathered} \frac{d}{{d\tau }}=\frac{\partial }{{\partial {\tau _0}}}+\varepsilon \frac{\partial }{{\partial {\tau _1}}}+{\varepsilon ^2}\frac{\partial }{{\partial {\tau _2}}}, \hfill \\ \frac{{{d^2}}}{{d{\tau ^2}}}=\frac{{{\partial ^2}}}{{\partial \tau _{0}^{2}}}+2\varepsilon \frac{{{\partial ^2}}}{{\partial {\tau _0}\partial {\tau _1}}}+{\varepsilon ^2}(\frac{{{\partial ^2}}}{{\partial \tau _{1}^{2}}}+2\frac{{{\partial ^2}}}{{\partial {\tau _0}\partial {\tau _2}}})+O({\varepsilon ^3}). \hfill \\ \end{gathered}$$

The previous operators clearly show that ${\varepsilon ^3}$ terms and higher are not taken into consideration. Equations (5)-(7) yield the following sets of equations when (9)-(12) are substituted. The various powers of $\varepsilon ,$ these equations are linked to the following

**(*i*) Order of** $$\varepsilon$$

13 $$\frac{{{\partial ^2}{\chi _{20}}}}{{\partial \tau _{0}^{2}}}+(1+\lambda )({\chi _{20}} - {\chi _{10}})=0,$$

14 $$\lambda \,{\chi _{20}} - (1+\lambda ){\chi _{10}}=0,\,$$

15 $$\frac{{\partial {v_0}}}{{\partial {\tau _0}}}+\frac{{{v_0}}}{{{{\tilde {c}}_p}{{\tilde {R}}_p}\,\omega }}=\frac{{\ell \,\tilde {\gamma }}}{{{{\tilde {c}}_p}}}\frac{{\partial {\chi _{20}}}}{{\partial {\tau _0}}}.$$

**(*ii*) Order of** ${\varepsilon ^2}$

16 $$\frac{{{\partial ^2}{\chi _{21}}}}{{\partial \tau _{0}^{2}}}+(1+\lambda )({\chi _{21}} - {\chi _{11}})= - {\tilde {\alpha }_2}(1+\lambda ){({\chi _{20}} - {\chi _{10}})^3} - 2\frac{{{\partial ^2}{\chi _{20}}}}{{\partial {\tau _0}\partial {\tau _1}}},$$

17 $$\lambda \,{\chi _{21}} - (1+\lambda ){\chi _{11}}=\lambda \,{\tilde {\alpha }_2}{({\chi _{10}} - {\chi _{20}})^3}+{\tilde {\alpha }_1}\chi _{{10}}^{3},$$

18 $$\frac{{\partial \,{v_1}}}{{\partial {\tau _0}}}+\frac{{{v_1}}}{{{{\tilde {c}}_p}{{\tilde {R}}_p}\,\omega }}=\frac{{\ell \,\tilde {\gamma }}}{{{{\tilde {c}}_p}}}(\frac{{\partial {\chi _{20}}}}{{\partial {\tau _1}}}+\frac{{\partial {\chi _{21}}}}{{\partial {\tau _0}}}) - \frac{{\partial {v_0}}}{{\partial {\tau _1}}}.$$

**(*iii*) Order of** ${\varepsilon ^3}$

19 $$\begin{gathered} \frac{{{\partial ^2}{\chi _{22}}}}{{\partial \tau _{0}^{2}}}+(1+\lambda )({\chi _{22}} - {\chi _{12}})=\frac{1}{2}\tilde {f}\,{e^{i\,p\,{\tau _0}}} - \tilde {G}\frac{{{\chi _{20}}}}{{\partial {\tau _0}}}+3\,{{\tilde {\alpha }}_2}(1+\lambda ){({\chi _{10}} - {\chi _{20}})^3}({\chi _{11}} - {\chi _{21}}) \hfill \\ \,\,\,\,\,\,\,\,\,\,\,\,\,\, - \tilde {c}\frac{{{\chi _{20}}}}{{\partial {\tau _0}}} - 2\frac{{{\partial ^2}{\chi _{20}}}}{{\partial {\tau _0}\partial {\tau _2}}} - 2\frac{{{\partial ^2}{\chi _{21}}}}{{\partial {\tau _0}\partial {\tau _1}}} - \frac{{{\partial ^2}{\chi _{20}}}}{{\partial \tau _{1}^{2}}} - \tilde {\gamma }\,{v_0}, \hfill \\ \end{gathered}$$

20 $$\lambda \,{\chi _{22}} - (1+\lambda ){\chi _{12}}=3\,\lambda \,{\tilde {\alpha }_2}{({\chi _{10}} - {\chi _{20}})^3}({\chi _{11}} - {\chi _{21}})+3\,{\tilde {\alpha }_1}\,\chi _{{10}}^{2}\,{\chi _{11}},$$

21 $$\frac{{\partial {v_2}}}{{\partial {\tau _0}}}+\frac{{{v_2}}}{{{{\tilde {c}}_{_{p}}}{{\tilde {R}}_p}\,\omega \,}}=\frac{{\ell \,\tilde {\gamma }}}{{{{\tilde {c}}_p}}}(\frac{{\partial {\chi _{22}}}}{{\partial {\tau _0}}}+\frac{{\partial {\chi _{21}}}}{{\partial {\tau _1}}}+\frac{{\partial {\chi _{20}}}}{{\partial {\tau _2}}}) - \frac{{\partial {v_1}}}{{\partial {\tau _1}}} - \frac{{\partial {v_0}}}{{\partial {\tau _2}}}.$$

It is possible to obtain the solutions of (13)-(21) in a serial way, which accentuates the solutions represented by the first system of Eqs. (13)-(15). Therefore, the overall solutions to the earlier equations (13) through (15) are as follows

22 $${\chi _{20}}\,=E\,{e^{i{\tau _0}}}+\bar {E}\,{e^{ - i{\tau _0}}},$$

23 $${\chi _{10}}=\frac{\lambda }{{1+\lambda }}(E\,{e^{i\,{\tau _0}}}+\bar {E}\,{e^{ - i\,{\tau _0}}}),$$

24 $${v_0}={\tilde {R}_p}\ell \tilde {\gamma }\,\omega \{ \frac{{E\,{e^{i{\tau _0}}}}}{{{{\tilde {R}}_p}{{\tilde {c}}_p}\omega - i}}+\frac{{\bar {E}\,{e^{ - i{\tau _0}}}}}{{{{\tilde {R}}_p}{{\tilde {c}}_p}\omega +i}}\} .$$

Here, E portrays abstruse complex processes occurring at sluggish time intervals ${\tau _a};\,\,(a=1,2)$, while $\bar {E}$ show its complex conjugate. To solve the higher order partial differential Eqs. (16)-(18), we can substitute the previously established solutions (22)-(24). The following solvability conditions are considered to obtain the secular terms,

25 $$2\,i\frac{{\partial E}}{{\partial {\tau _1}}}+3{E^2}\bar {E}\frac{{({{\tilde {\alpha }}_1}{\lambda ^3}+1)}}{{{{(1+\lambda )}^3}}}=0.\,$$

Therefore, the following equations represent the second-order approximation

26 $${\chi _{21}}=\frac{{{{\tilde {\alpha }}_1}\,{B^3}}}{8}{(\frac{\lambda }{{1+\lambda }})^3}\,{e^{3\,i\tau \,}}+\frac{{\,{B^3}}}{{8{{(1+\lambda )}^3}}}\,{e^{3\,i\tau }}+CC,\,$$

27 $$\begin{gathered} {\chi _{11}}=\frac{1}{{1+\lambda }}[\frac{{{{\tilde {\alpha }}_1}\lambda \,}}{8}{(\frac{\lambda }{{1+\lambda }})^3}\,{E^3}{e^{3\,i\tau \,}}+\frac{\lambda }{{8{{(1+\lambda )}^3}}}{E^3}{e^{3\,i\,\tau }}+(\frac{{\lambda {{\tilde {\alpha }}_2}}}{{{{(1+\lambda )}^3}}} - {{\tilde {\alpha }}_1}{(\frac{\lambda }{{1+\lambda }})^3})({E^3}{e^{3\,i\tau \,}} \hfill \\ \,\,\,\,\,\,\,\,+3{E^2}\bar {E}{e^{\,i\tau \,}})]+CC,\, \hfill \\ \end{gathered}$$

28 $$\begin{gathered} {v_1}\,=\frac{{3{{\tilde {c}}_p}{{\tilde {R}}_p}}}{{2(1 - i{{\tilde {c}}_p}{{\tilde {R}}_p}\omega )}}(\frac{{\tilde {\gamma }\omega \ell }}{{{{\tilde {c}}_p}}}+\frac{{\tilde {\gamma }\ell \omega {{\tilde {R}}_p}}}{{{{\tilde {c}}_p}{{\tilde {R}}_p}\omega - i}})[\frac{{1+{\alpha _1}{\lambda ^3}}}{{{{\left( {1+\lambda } \right)}^3}}}]{E^2}\bar {E}{e^{i{\tau _0}}}+\frac{{3\tilde {\gamma }\,\omega \,\ell }}{{8{{\tilde {c}}_p}}}[{\alpha _1}\frac{{{\lambda ^3}}}{{{{(1+\lambda )}^3}}} \hfill \\ \,\,\,\,\,+\frac{1}{{{{(1+\lambda )}^3}}}]\frac{{{{\tilde {c}}_p}{{\tilde {R}}_p}\omega }}{{(3{{\tilde {c}}_p}{{\tilde {R}}_p}\omega - i)}}{E^3}{e^{3i{\tau _0}}}+CC\,, \hfill \\ \end{gathered}$$

where $CC$ represents the previous solutions’ conjugations. In the context of the above, the below expressions lead to the elimination of the following terms

29 $$\begin{gathered} - 2i\frac{{\partial E}}{{\partial {\tau _2}}}+6\,i{E^2}\bar {E}\frac{{({{\tilde {\alpha }}_1}{\lambda ^3}+1)}}{{{{(1+\lambda )}^3}}} - i(\,\tilde {c}+\tilde {G})E+\frac{3}{8}{\alpha _2}{E^2}\bar {E}[{\lambda ^3}{\alpha _1}(\lambda - 2)+8\lambda {\alpha _2} \hfill \\ +\lambda - 1]/{(1+\lambda )^5} - \frac{{E\tilde {\gamma }{{\tilde {R}}_p}\tilde {\mu }\omega \ell }}{{{{\tilde {R}}_p}{{\tilde {c}}_p}\omega - i}} - \frac{{9{{\tilde {\alpha }}_1}{\lambda ^2}}}{{{{(1+\lambda )}^2}}}{E^2}\bar {E}=0. \hfill \\ \end{gathered}$$

In the last step, we derive the third-order solution as shown below

30 $$\begin{gathered} {\chi _{22}}=\frac{{\tilde {f}\,{e^{i\,p\,{\tau _0}}}}}{{2(1 - p)}}\,+\frac{1}{{64{{(1+\lambda )}^6}}}{E^3}{e^{3\,\,{\tau _0}}}[{H_1}+E\,{H_2}\,{{\tilde {\alpha }}_2}+6\,E\,\bar {E}(3+3\,i\,{\lambda ^6}\tilde {\alpha }_{1}^{2} \hfill \\ \,\,\,\,\,\,\,\,+{{\tilde {\alpha }}_2}{H_3}+{H_4})]+CC, \hfill \\ \end{gathered}$$

31 $$\begin{gathered} {\chi _{12}}=\frac{1}{{1+\lambda }}\{ \lambda \,{\chi _{12}} - [{\lambda ^4}(E\,{e^{2\,i\,{\tau _0}}}(\lambda - 8) - 24\,\bar {E})\tilde {\alpha }_{1}^{2}+{{\tilde {\alpha }}_2}{H_6}+(E\,{e^{2\,i\,{\tau _0}}}{(1+\lambda )^2} \hfill \\ \,\,\,\,\,\,\,\,+{H_7}{{\tilde {\alpha }}_2}){\lambda ^2}{{\tilde {\alpha }}_1}]{H_5}\} +CC, \hfill \\ \end{gathered}$$

32 $$\begin{gathered} {v_2}\,={E^3}{H_9}[\frac{{{{\tilde {R}}_p}\omega \ell \tilde {\gamma }{H_8}}}{{(1+3i\,{{\tilde {c}}_p}{{\tilde {R}}_p}\omega )}}{e^{3\,\,{\tau _0}}} - 8\lambda (\frac{{5{{\tilde {R}}_p}\omega \ell \tilde {\gamma }E}}{{(1+5i\,{{\tilde {c}}_p}{{\tilde {R}}_p}\omega )}}{e^{5\,\,i\,{\tau _0}}}+\frac{{27\lambda +27}}{{(1+3i\,{{\tilde {c}}_p}{{\tilde {R}}_p}\omega )}} \hfill \\ \,\,\,\,\, \times {{\tilde {R}}_p}\omega \ell \tilde {\gamma }{e^{3\,\,{\tau _0}}}){{\tilde {\alpha }}_2}++6\,E\,\bar {E}(3+3\,i\,{\lambda ^6}\tilde {\alpha }_{1}^{2}+3{{\tilde {\alpha }}_2}{H_3}+3{H_4})\frac{{{{\tilde {R}}_p}\omega \ell \tilde {\gamma }}}{{(1+3i\,{{\tilde {c}}_p}{{\tilde {R}}_p}\omega )}}] \hfill \\ \,\,\,\,\, - \frac{9}{8}{H_{10}}]\frac{{3\tilde {\gamma }\,\omega \,\ell }}{{8{{\tilde {c}}_p}}}[{\alpha _1}\frac{{{\lambda ^3}}}{{{{(1+\lambda )}^3}}}+\frac{1}{{{{(1+\lambda )}^3}}}]{H_{11}}+CC\,, \hfill \\ \end{gathered}$$

where ${H_j}\,(j=1,2,3,\cdots,11)$ are formulated as in Appendix (I).

Finally, Different methods can be used to analyze the nonlinear oscillations of a system with a series spring, piezoelectric device, and feedback controller. Below in Table (1) a comparison of the MSM with other common solution techniques:

**Table 1:** Shows a comparison of the multiple scale method with other solution methods.

Method	Advantages	Disadvantages
Multiple Scale Method (MSM)	- Handles weak nonlinearity and slow/fast time scales effectively. - Avoids secular terms that cause divergence in perturbation methods. - Provides analytical insight into resonance and bifurcation behavior.	- Limited to weakly nonlinear systems. - Approximate method, accuracy depends on order of expansion.
Numerical Methods (e.g., Runge-Kutta, Finite Difference)	- Can handle strongly nonlinear and chaotic systems. - Provides highly accurate solutions without assumptions.	- Computationally expensive for long-term analysis. - Lacks explicit analytical expressions.
Harmonic Balance Method	- Effective for periodic steady-state solutions. - Useful in forced vibration analysis.	- May fail for complex transient dynamics. - Requires truncation of higher harmonics for practical use.
Perturbation Methods (e.g., Lindstedt-Poincaré, Method of Averaging)	- Simple and effective for weakly nonlinear systems. - Provides analytical approximations.	- Leads to secular terms if not handled properly. - Limited to small perturbations.
Variational Methods (e.g., Ritz Method, Energy Balance Method)	- Provides approximate solutions based on energy considerations. - Useful for conservative nonlinear systems.	- Accuracy depends on the choice of trial functions. - Limited applicability to dissipative systems.

## Vibration at resonance

In the current section, the resonance vibrations are investigated. These occur when the denominators of the last two approximations are set to zero. Therefore, the latter can be obtained from the higher approximations when the external force’s frequency is in close proximity to the natural frequency of the simulated linearized system; the main resonance occurs in the system at $p \approx 1$. To study this situation, we assume the detuning parameter $\sigma$ as follows

33 $$p=1+\sigma$$

Another way to consider the detuning parameter is the distance from strict resonance and oscillation. By removing the secular term, we obtain the condition for solvability. Therefore, the following equation calculates the probability of satisfying the condition

34 $$\begin{gathered} \frac{1}{2}f\,{e^{i\,{\tau _0}\,\sigma }} - 2i\frac{{\partial E}}{{\partial {\tau _2}}}+6\,i\,{E^2}\bar {E}\frac{{({{\tilde {\alpha }}_1}{\lambda ^3}+1)}}{{{{(1+\lambda )}^3}}} - i(\,\tilde {c}+\tilde {G})E+\frac{3}{8}{\alpha _2}{E^2}\bar {E}[{\lambda ^3}{\alpha _1}(\lambda - 2)+8\lambda {\alpha _2} \hfill \\ +\lambda - 1]/{(1+\lambda )^5} - \frac{{E\tilde {\gamma }{{\tilde {R}}_p}\tilde {\mu }\omega \ell }}{{{{\tilde {R}}_p}{{\tilde {c}}_p}\omega - i}} - \frac{{9{{\tilde {\alpha }}_1}{\lambda ^2}}}{{{{(1+\lambda )}^2}}}{E^2}\bar {E}=0. \hfill \\ \end{gathered}$$

The functions E can be ascertained as in the earlier solvability conditions. Consequently, it can be shown in a polar form as follows

35 $$E=\frac{{\tilde {a}}}{2}{e^{i\,\Psi }};\,\,\,\,\,a=\varepsilon \tilde {a}.$$

In this case, $\tilde {a}$ and $\psi$ stand for the solution’s amplitude and phase, respectively. The phase has been modified as follows

36 $$\theta ={\tau _1}\,\tilde {\sigma } - \psi ,\,\,\,\,\,\,\sigma =\varepsilon \tilde {\sigma }.$$

The system below can be produced by splitting the real and imaginary components based on the previous and inserting (35)-(36) into (34).

37 $$\begin{gathered} a\frac{{d\theta }}{{d\tau }}=\frac{f}{2}\cos \theta +a\,\sigma +{H_{13}}a+\frac{{3({\alpha _2}+{\lambda ^3}{\alpha _1})}}{{8{{(1+\lambda )}^3}}}{a^3} - {H_{12}}{a^5}, \hfill \\ \frac{{d\,a}}{{d\tau }}=\frac{f}{2}\sin \theta - {H_{14}}a - \frac{{(c+G)}}{2}a. \hfill \\ \end{gathered}$$

Appendix (I) contains the expressions for ${H_{12}},{H_{13}},$and ${H_{14}}$. The RK- 4 method can be used to solve the above modulation system (37) numerically and show the functions time-dependent behavior and their associated phase planes as well as at resonance both with and without control. Therefore, the following data and initial conditions are taken into account

$$\begin{gathered} c=0.001,\,\,\,\,\,\,\,\,\,{\alpha _1}=1.025,\,\,\,\,\,\,\,\,\,\,\lambda =0.9,\,\,\,\,\,{f_1}=0.02,\,\,\,\,\,{\alpha _2}=1.1,\, \hfill \\ \gamma =0.1,\,\,\,\,\,\,\sigma =0.01,\,\,\,\,\,\,\,\,\,\,Rp=500,\,\,\,\,\,\,\,\,\,\,cp=10,\,\,\,\,\,\,\,\,\,\,p=0.005. \hfill \\ \end{gathered}$$

**Fig. 3:**
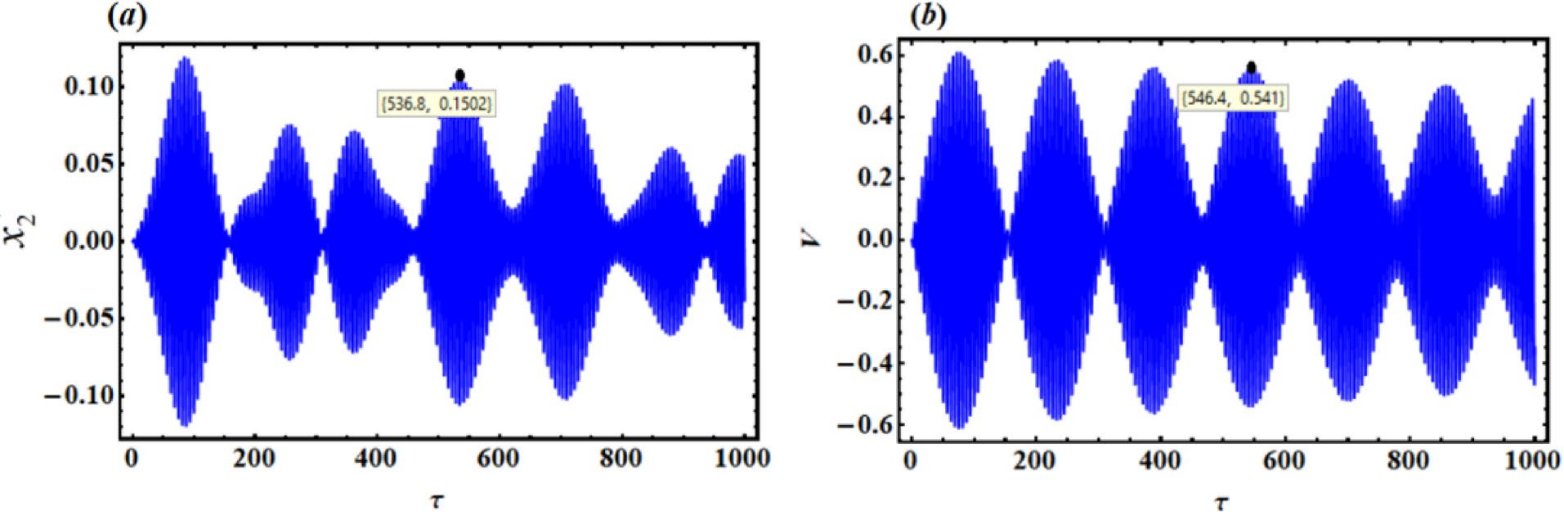
Time histories ${x_2}$and V without resonance or control ($G=0$).

**Fig. 4:**
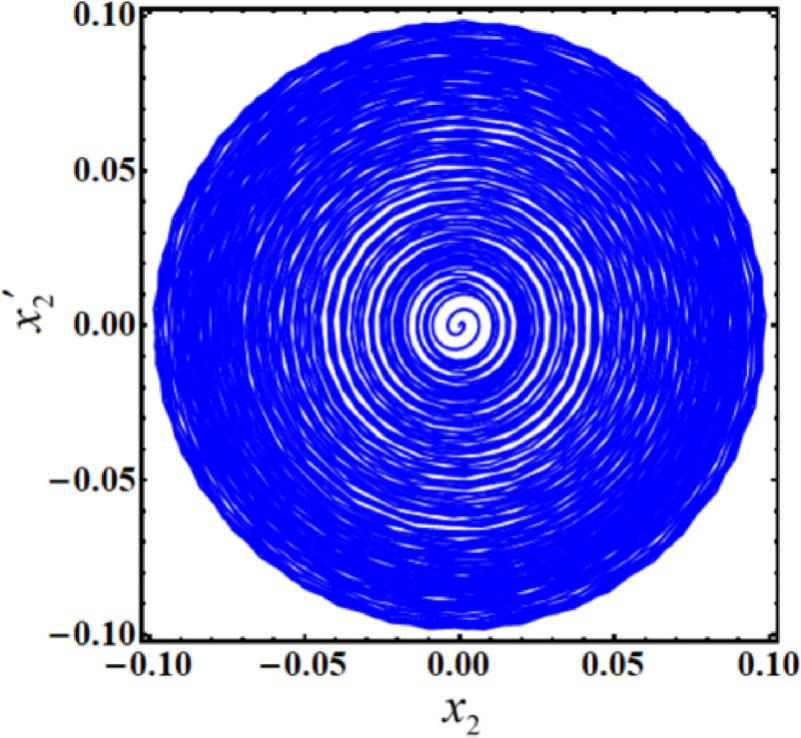
The PP ${x_2}\,{x^{\prime}_2}$ without resonance or control ($G=0$).

**Fig. 5:**
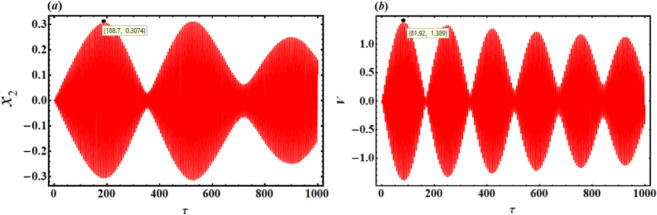
Time histories ${x_2}$ and V with resonance case $p \approx 1$without control ($G=0$).

**Fig. 6:**
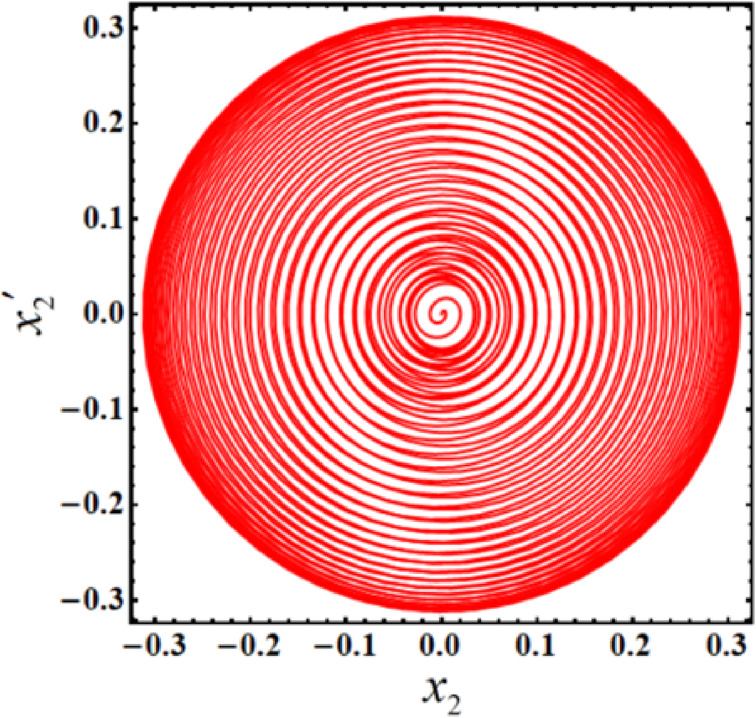
The PP ${x_2}\,{x^{\prime}_2}$ with resonance case $p \approx 1$without control ($G=0$).

**Fig. 7:**
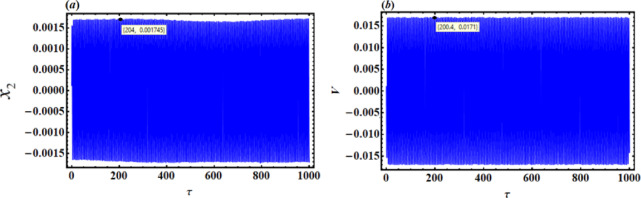
Time response solutions ${x_2}$ and V with resonance case $p \approx 1$with control ($G=5$).

**Fig. 8:**
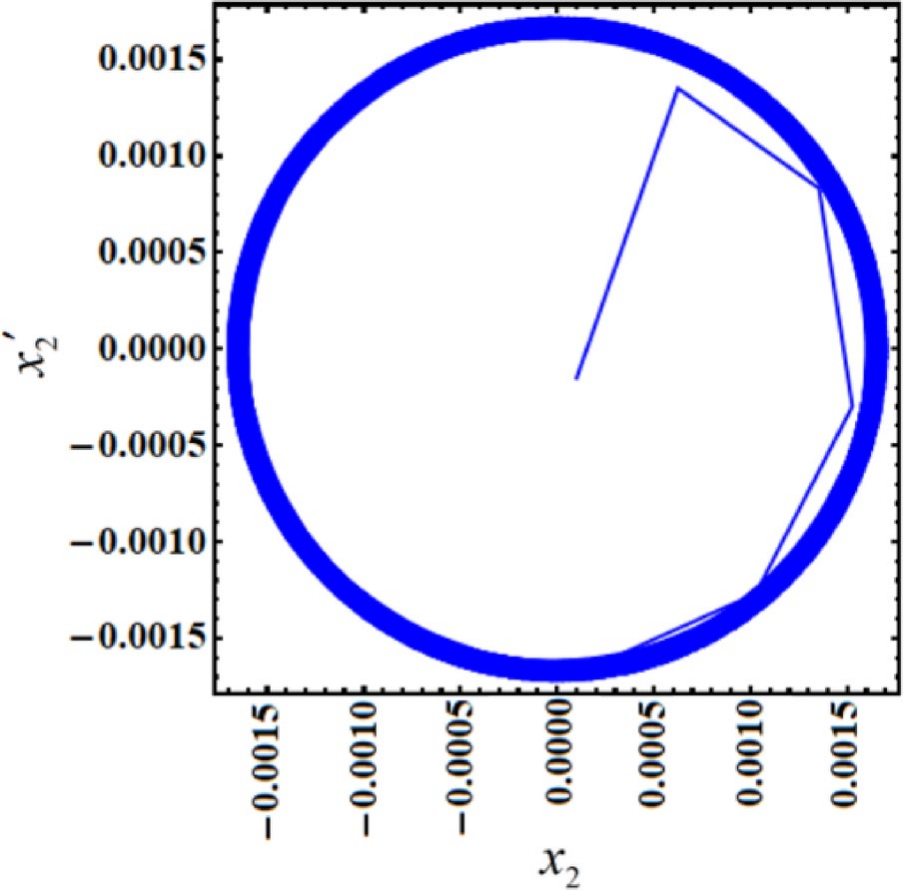
The PP ${x_2}\,{x^{\prime}_2}$ with resonance case $p \approx 1$without control ($G=5$).

**Fig. 9:**
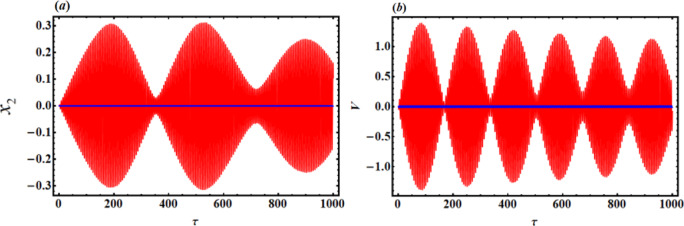
Time response solutions ${x_2}$ and V with resonance case $p \approx 1$ with and without control represented by the blue and the red curves, respectively.

**Fig. 10:**
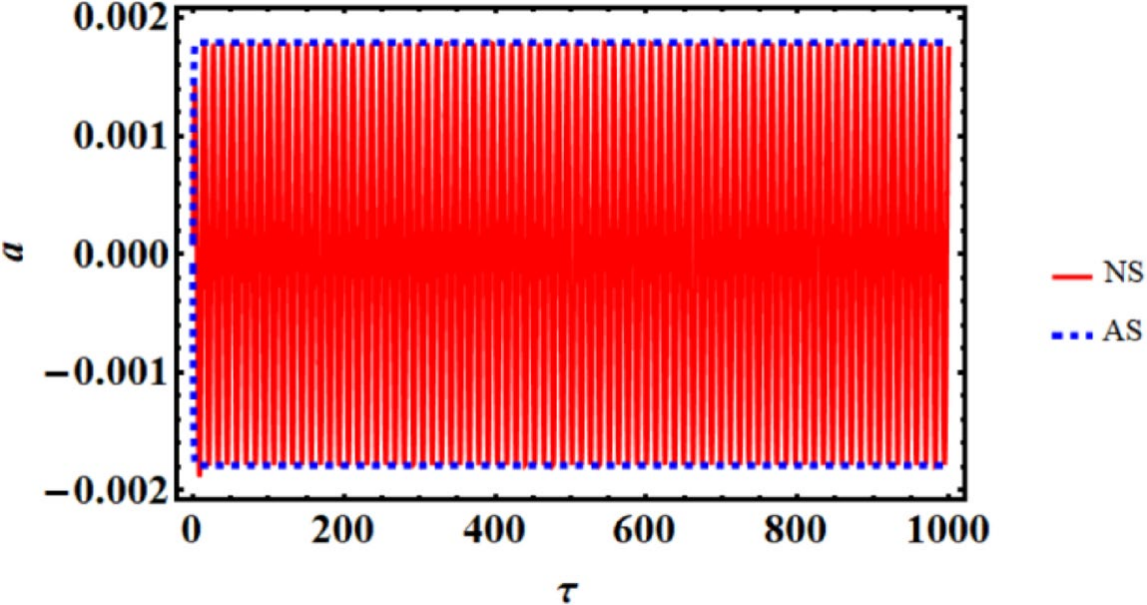
The evaluation of AS and NS.

**Fig. 11:**
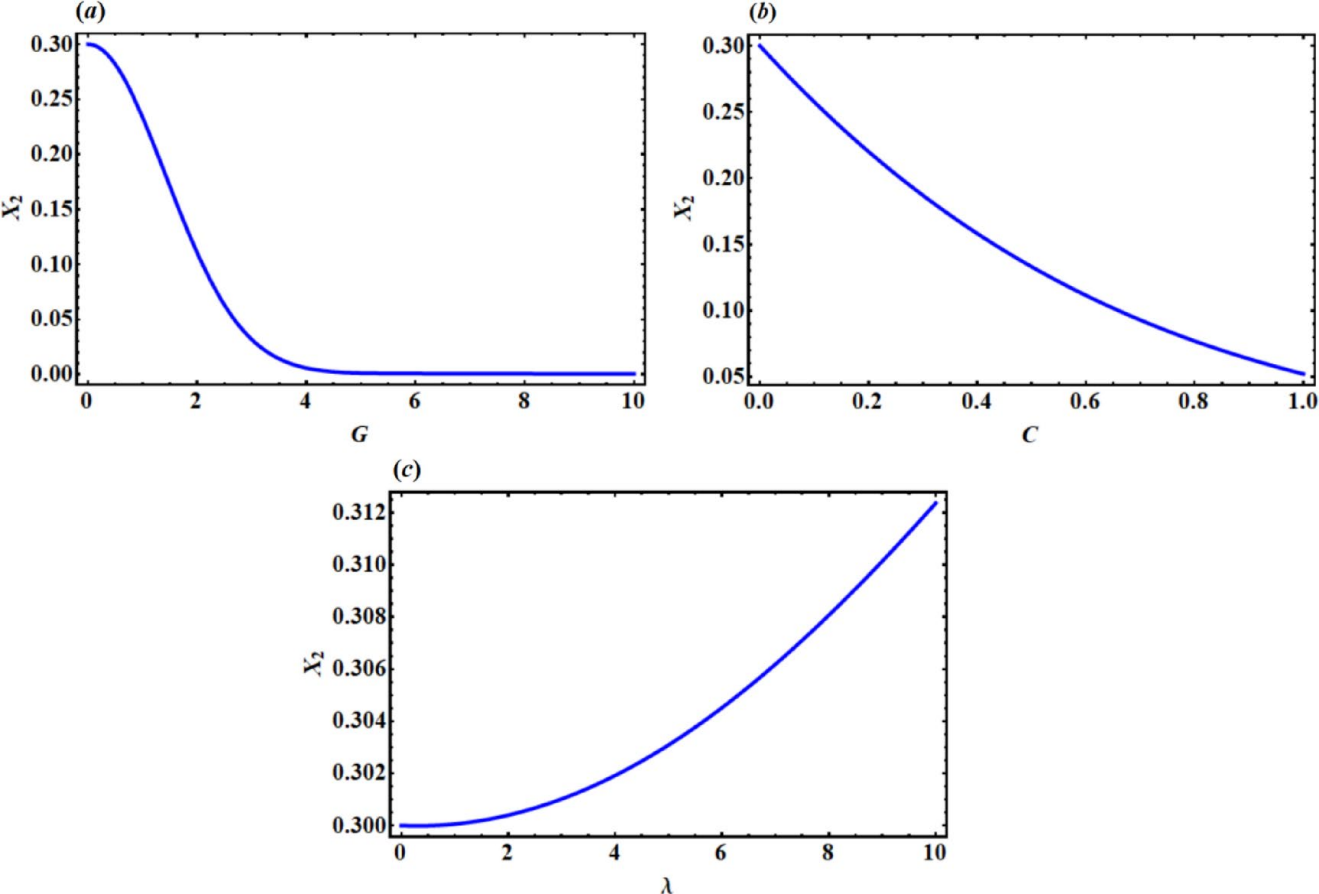
The impact of system parameters G and C on the amplitude ${x_2}$.

**Fig. 12:**
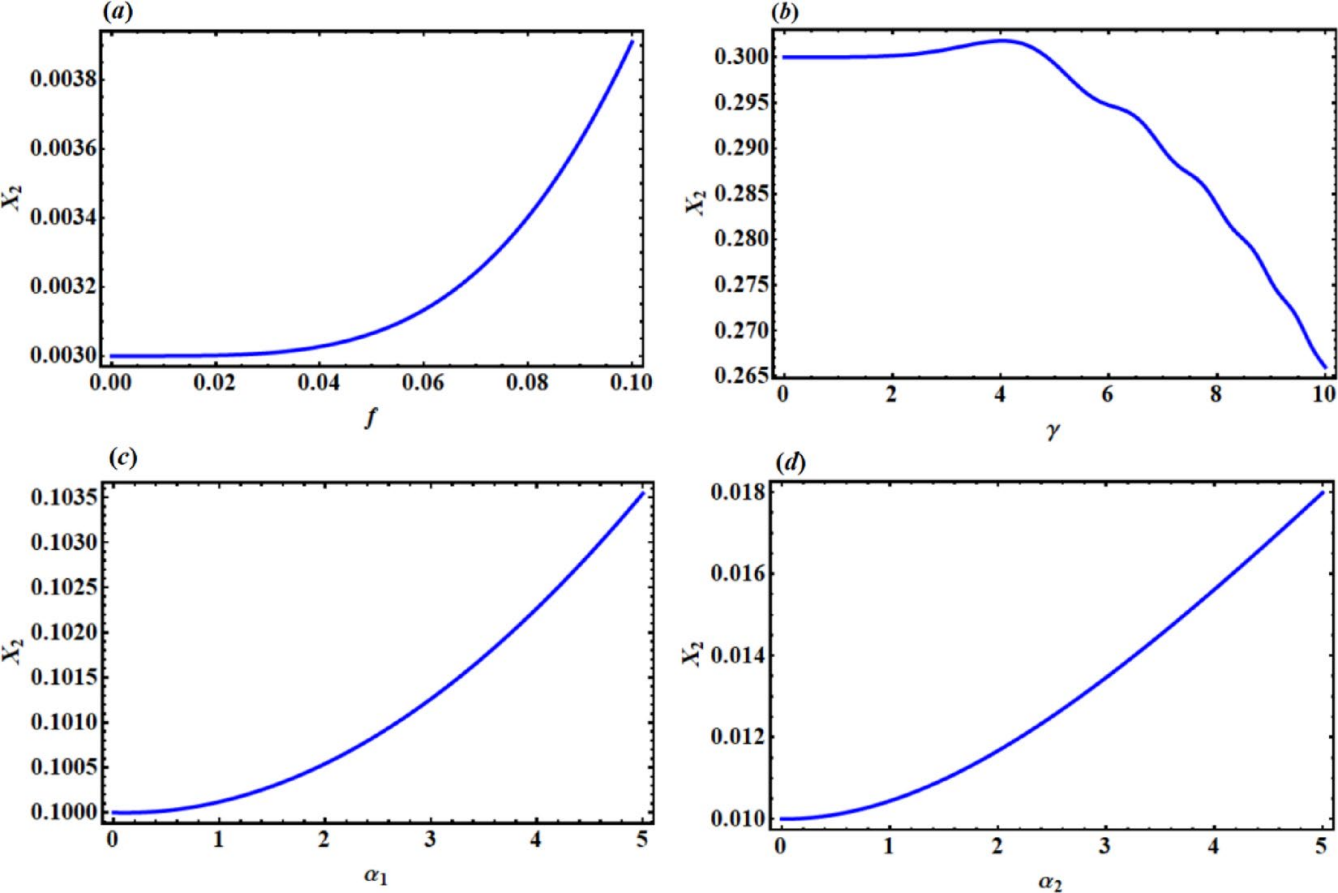
The influence of system parameters f and $\gamma$ on the amplitude ${x_2}$.

As a result, Figs. 3,4,5,6,7,8,9,10,11,12 are plotted. In this context, the curves in Figs. 3 and 4 represent the time history of the waves for the functions $({x_2},\,V)$ and the phase portrait (PP) of ${x_2}$, respectively, in the case of without resonance and control. We observe that the amplitudes of these waves are $0.1502$ and $0.541$, and the curves in Figs. 5 and 6 illustrate the time history of the waves at resonance without control (i.e., $G=0$). It is noted that the amplitudes of these waves are $0.3074$ and $1.389$, suggesting a risk to the system, underscoring the need for control measures to minimize and counteract these harmful vibrations and maintain system integrity.

In contrast, Figs. 7 and 8 show the time histories and PP of the same functions at resonance with the NVF controller. It is noteworthy that the magnitudes’ values have been reduced to $0.001745$ and $0.0171$. This was basically explained by comparing with and without control, as Fig. 9 illustrates. Through the influence of the NVF controller, we found that the amplitude of ${x_2}$ decreased by 99.41%, while the amplitude of V decreased by 98.76%. This is beneficial as the primary goal of control is to minimize dynamic oscillations. The curves in Fig. 10 highlight a high level of agreement between AS and NS, reinforcing the accuracy of MSM.

Allow us to demonstrate the change in the magnitude of ${x_2}$ when changing the system parameters $G,C,\,\lambda ,f,\,\gamma ,\,{\alpha _1},\,$and ${\alpha _2}$. It is important to note that the magnitude decreases with the increase in the values ​​of G and C in Fig. 11(a, b), whereas the magnitude ${x_2}$ increases with the increase of $\lambda$ as illustrated in Fig. 11c. In addition, the system amplitude is inversely proportional to the values of $f,\,{\alpha _1},\,$ and ${\alpha _2}$ as shown in Fig. 12(a, c,d), respectively. It is observed that the magnitude is virtually constant with the increase in the value $\gamma$ until a certain value, the system collapses with the increase in the value of $\gamma$, in which it decreases with the increase in the value ​​of $\gamma$, as demonstrated in Fig. 12 b.

## Steady-state strategy

This section studies the steady-state solution of the dynamic system being analyzed. In this case, we consider the null value of of $\frac{{da}}{{d\tau }}=\frac{{d\theta }}{{d\tau }}=0$^36^. Therefore, we can create the subsequent algebraic system of equations based on equations (37)

38 $$\begin{gathered} \frac{f}{2}\cos \theta +a\,\sigma +{H_{13}}a+\frac{{3({\alpha _2}+{\lambda ^3}{\alpha _1})}}{{8{{(1+\lambda )}^3}}}{a^3} - {H_{12}}{a^5}=0, \hfill \\ \frac{f}{2}\sin \theta - {H_{14}}a - \frac{{(c+G)}}{2}a=0. \hfill \\ \end{gathered}$$

The following frequency response equations are obtained from an analysis of the final system (38) after the changed phase $\theta$is removed^37^

39 $${f^2}=4{(a\,\sigma +{H_{13}}a+\frac{{3({\alpha _2}+{\lambda ^3}{\alpha _1})}}{{8{{(1+\lambda )}^3}}}{a^3} - {H_{12}}{a^5})^2}+4{({H_{14}}a+\frac{{(c+G)}}{2}a)^2}.$$

By looking at slight variations in amplitude and phase, we will examine its behavior close to the fixed points.

40 $$a={a_0}+{a_1},\,\,\,\,\,\,\,\,\theta ={\theta _0}+{\theta _1},$$

where ${a_0}$and ${\theta _0}$denotes the steady-state solutions, while ${a_1}$ and ${\theta _1}$denotes the minor disturbances in contrast to ${a_0}$ and ${\theta _0}$. The following system can be obtained after linearization by substituting (40) into (37).

41 $$\begin{gathered} {a_0}\frac{{d{\theta _1}}}{{d\tau }}= - \frac{f}{2}\sin {\theta _0}{\theta _1}+{a_1}\,\sigma +{H_{13}}{a_1}+\frac{{3({\alpha _2}+{\lambda ^3}{\alpha _1})}}{{8{{(1+\lambda )}^3}}}a_{0}^{2}{a_1} - 5{H_{12}}a_{0}^{4}{a_1}, \hfill \\ \frac{{d{a_1}}}{{d\tau }}=\frac{f}{2}\cos {\theta _0}{\theta _1} - {H_{14}}{a_1} - \frac{{(c+G)}}{2}{a_1}. \hfill \\ \end{gathered}$$

This system’s solutions can be written as a linear combination of $\,{\zeta _b}\,{e^{\delta \tau }}$where${\zeta _b}\,\,(b=1,2)$ denotes constants and $\delta$denotes the perturbation’s eigenvalue. The modest perturbation functions served as its foundation${a_1}$ and ${\theta _1}$. Considering the aforementioned, In (41), the fixed points demonstrate asymptotic stability if the real portions of the characteristic equation’s roots are negative.

42 $${\delta ^2}+{\Gamma _1}\delta +{\Gamma _2}=0,$$

where

43 $$\begin{gathered} {\Gamma _1}=[f\sin {\theta _0}+(c+G+2{H_1}){a_0}]/2{a_0}, \hfill \\ {\Gamma _2}=f[(c+G+2{H_1})\sin {\theta _0} - 2({d_1}+{d_2}+\sigma )\cos {\theta _0}]/4{a_0}. \hfill \\ \end{gathered}$$

In which ${d_1}$and ${d_2}$ have expressions in Appendix (I)

For a fixed point to exhibit stability, it is essential that the following conditions, as outlined by the Routh–Hurwitz criterion^38^, are met.

44 $${\Gamma _{\text{1}}}>0,\,\,\,\,\,\,\,\,\,\,\,\,\,\,\,\,\,\,\,\,\,\,\,\,\,\,\,\,{\Gamma _{\text{2}}}>0.$$

It is important to note that the Routh-Hurwitz criteria are regarded as essential instruments in control theory and linear time-invariant system stability analysis. They offer a methodical approach to figuring out whether the roots of a polynomial equation are stable without having to do the calculations directly. Determining the stability of the system is the main reason these criteria are important. Additionally, by building and analyzing the Routh array, they enable a faster and simpler determination of stability rather than solving for the characteristic polynomial’s roots, which can be algebraically demanding. Furthermore, by simplifying the problem and breaking it down into manageable steps, the previously mentioned method can pinpoint the challenging and intricate foundations of higher-order systems.

## Examining stability

**Fig. 13:**
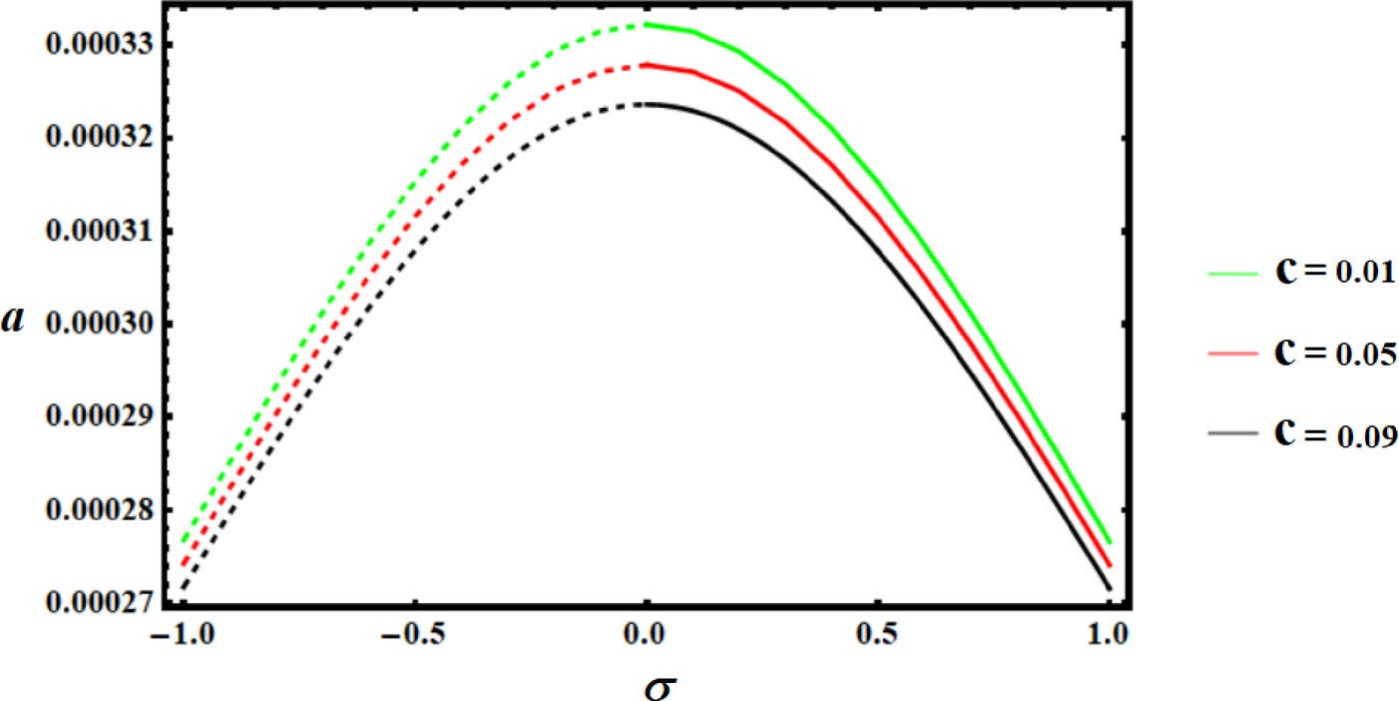
Amplitudes’ resonance curve *a *versus $\sigma$ at $c=0.01,0.05,0.09.$.

**Fig. 14:**
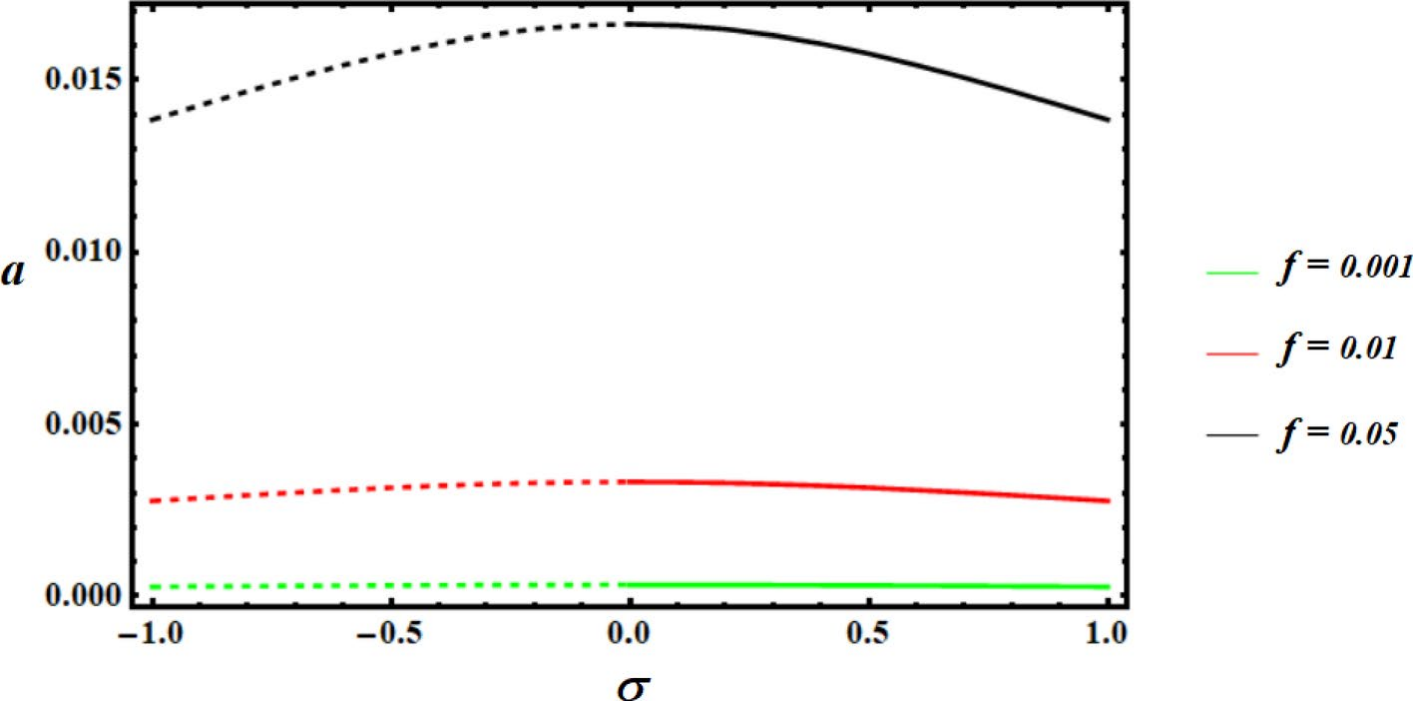
Amplitudes’ resonance curve *a *versus $\sigma$ at $f=0.001,0.01,0.05.$.

**Fig. 15:**
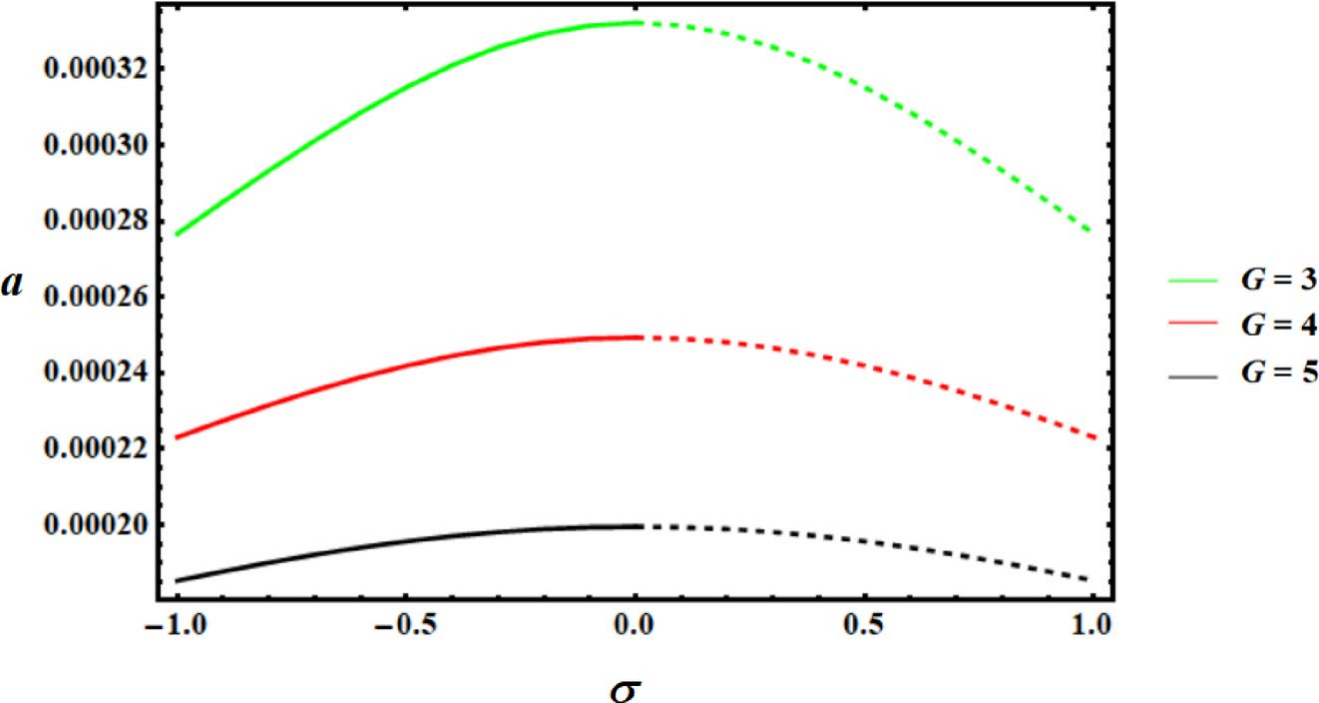
Amplitudes’ resonance curve* a* versus $\sigma$ at $G=3,4,5.$.

Using the nonlinear stability approach, this section seeks to evaluate the studied model’s nonlinear stability^39^. We will utilize the graphed curves of resonance response from the numerical solutions of (39) to show the stability regions of the fixed points, as illustrated in Figs. 13,14,15. The values of the used parameters are employed to generate these curves are as follows.

Keep in mind that the dashed curves indicate unstable fixed points, whereas the continuous curves signify stable fixed points. These numbers demonstrate the effectiveness of the applied control by showing that the stability zone grows as the control value does.

## The performance of the piezoelectric device

This section looks at how the piezoelectric device affects the behavior of the dynamical system and assesses how it contributes to the generation of electrical energy. The mechanical stress produced by the dynamic model’s vibration can cause the dielectric materials employed in this device to polarize. These materials polarize and produce an electrical field in our circumstances. As a result, mechanical energy is transformed into the required electrical energy using this device and the dynamical model. The electrical energy produced by the energy-collecting device has a wide range of applications, such as medical remote sensing, aerospace projects, emergency medical response monitoring, structural monitoring, and military use.

**Fig. 16:**
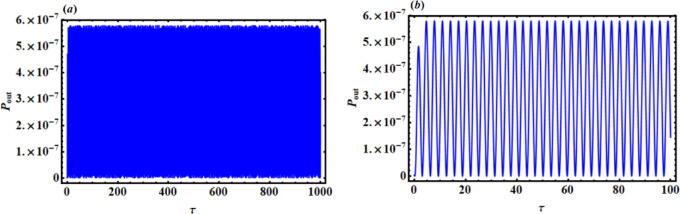
$${p_{out}}$$ versus time $$\tau$$ : (**a**) $$\tau =0:1000$$, (**b**) $$\tau =0:100$$.

**Fig. 17:**
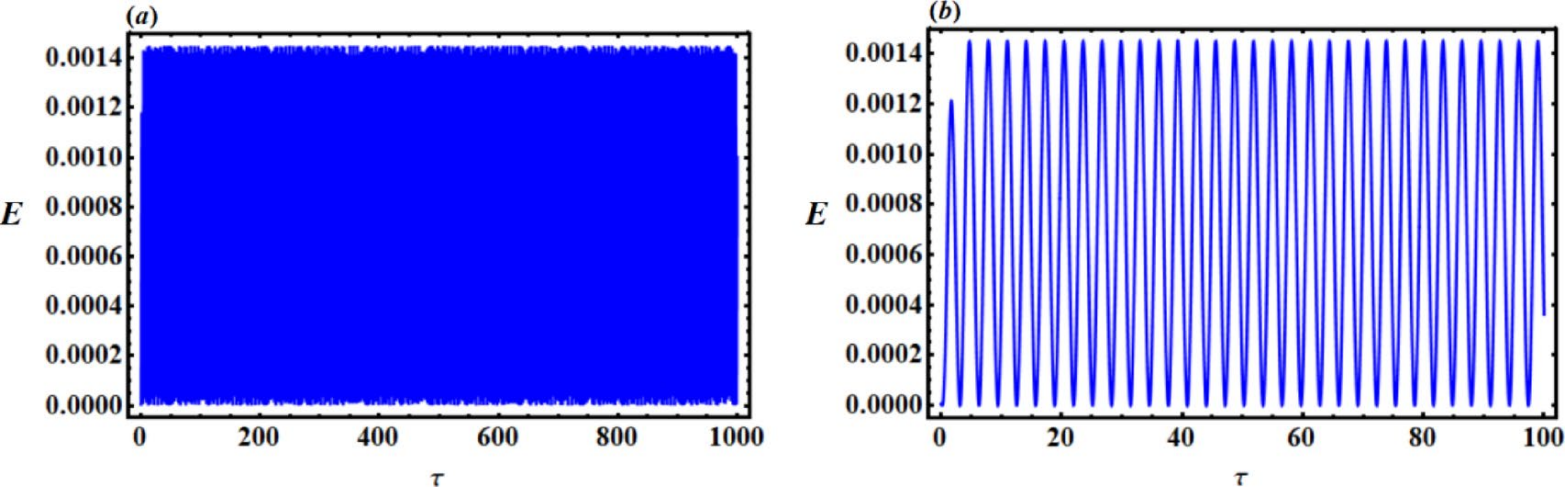
E versus time $$\tau$$ : (**a**) $$\tau =0:1000$$, (**b**) $$\tau =0:100$$.

Figures 16 and 17, respectively, display the piezoelectric’s powers ${p_{out}}$ and generated energy E with time part (*a*) $\tau =0:1000$but part (*b*) $\tau =0:100$. A closer inspection of certain regions of these images shows that standing waves have uniformly dissipated throughout the entire period.

## Bifurcation and the chaos analysis

Bifurcation often serves as a pathway to chaos^40,41^. As a system parameter changes, it may undergo period-doubling bifurcations, progressively leading to chaotic behavior. By studying bifurcations, we can anticipate when and how a system transitions into chaos, providing valuable insights into the emergence of complex dynamics.

**Fig. 18:**
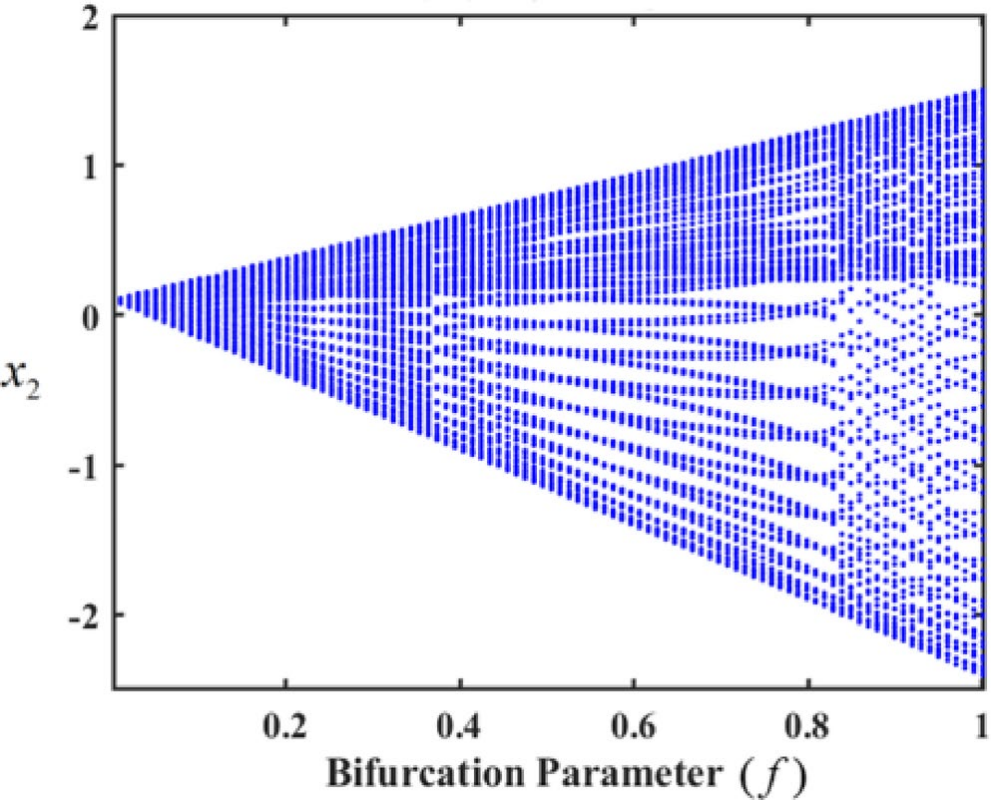
Bifurcation diagram of f at $p=0.05$.

In the analyzed system, the control gains significantly impact its behavior, giving rise to varied forms of motion. Bifurcation diagrams of ${x_2}$ a versus the excitation amplitude f were used to illustrate these motion types. Initially, we explore the system’s chaotic behavior in an uncontrolled state, as depicted in Fig. 18, which shows the bifurcation diagram of ${x_2}$. The figures reveal that the uncontrolled system exhibits two distinct complex behaviors over different ranges of f, with each range corresponding to a specific degree of chaotic motion.

**Fig. 19:**
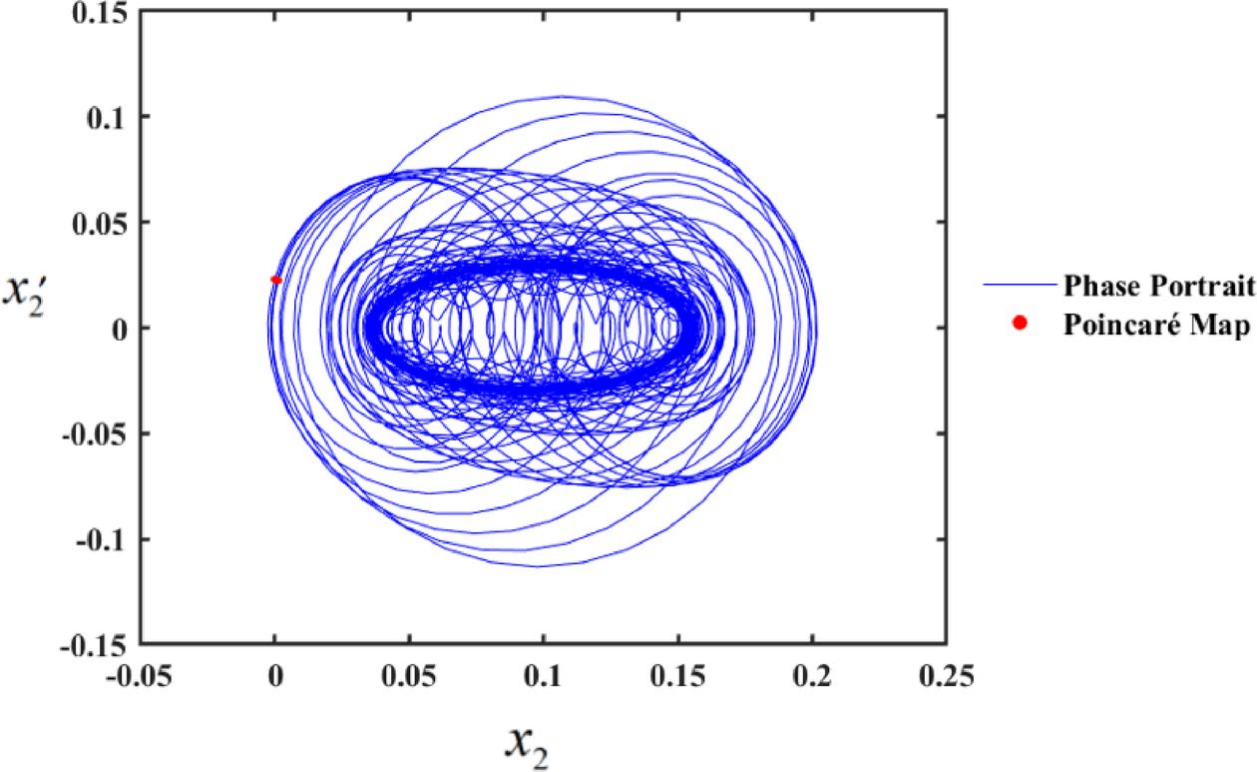
PP and PMs at $f=0.01$.

**Fig. 20:**
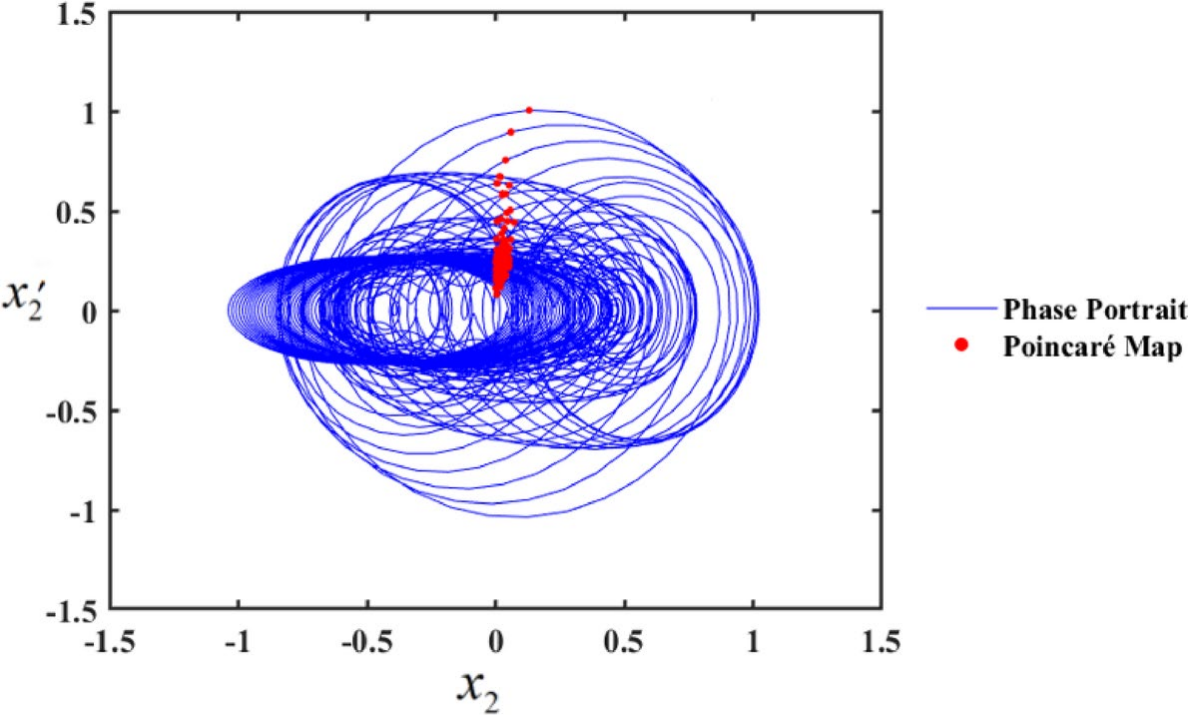
PP and PMs at $f=1$.

In the first range, at $f \in [0,0.01]$, the motion follows a periodic sequence. Conversely, in the second range, at $f \in [0.01,0.1]$, the system demonstrates chaotic behavior. To validate these findings, we simulated the PPs and PMs for various values of f. Specifically, at $f \in [0.1,1]$, the system displays hyper-chaotic behavior, whereas f results in a hyper-chaotic state, as illustrated in Figs. 19 and 20, respectively demonstrated in Fig. 18. The figures display varying values of the external excitation amplitude ${f_1}$. We focus on the value $f=0.01$ in Fig. 19.

One such red-dot pattern displayed by PMs closely resembles a closed curve of ${x_2},$ playing a role in the periodic motion of the system. The chaotic state that appears in the f values cause the red dots at $f=1$, as seen in Fig. 20, to manifest chaotic behavior. Notably, as f increases, the system’s complexity grows, transitioning from chaos to hyper-chaos. In the visualizations, the blue curves represent PPs, while the red dots correspond to the PMs.

**Fig. 21:**
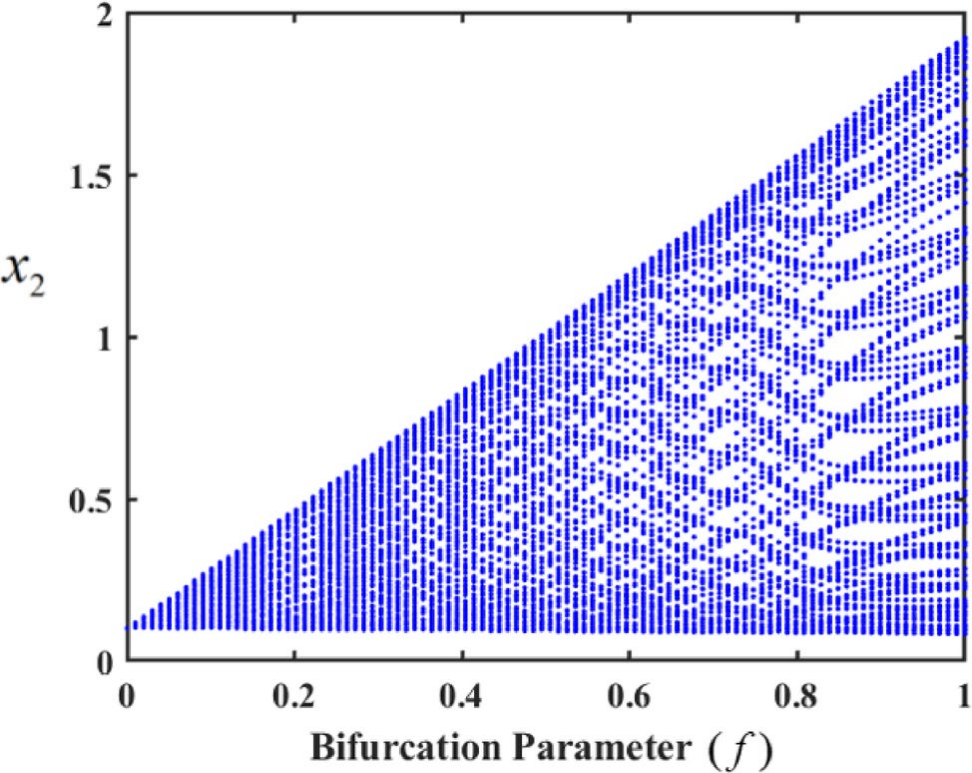
Bifurcation diagram of f at $G=\,5$.

**Fig. 22:**
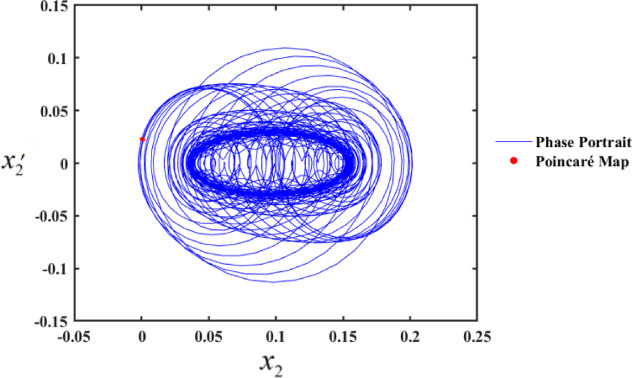
PP and PMs at $f=0.05$.

**Fig. 23:**
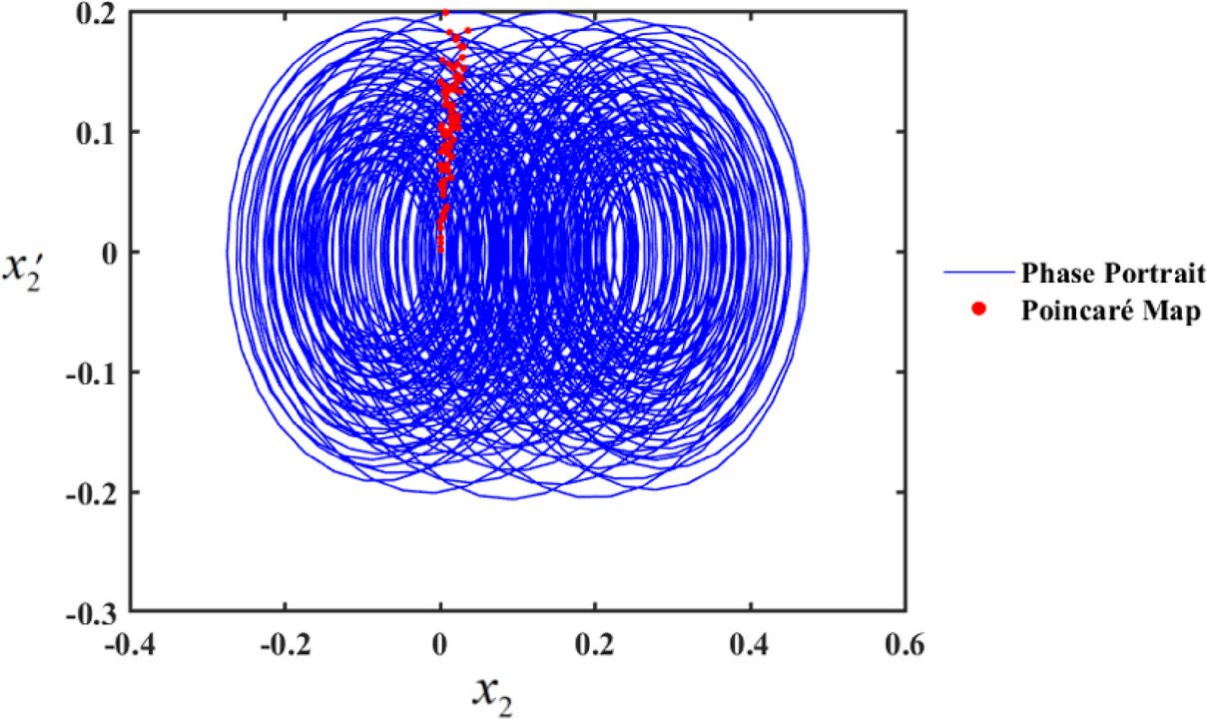
PP and PMs at $f=0.19$.

Figure 21 shows the same behavior in Fig. 18, but with control when $G=\,5$. Also, the PMs in Figs. 22 and 23 shows a chaotic response that confirms the system’s complexion. The chaotic response of a dynamical system and PMs provide valuable insights into the intricate dynamics of nonlinear systems. We conducted a bifurcation analysis to evaluate the controlled system’s behavior, as illustrated in the bifurcation diagram in Fig. 21 and supported by the PPs and PMs in Figs. 22 and 23. Our analysis shows that the control device greatly enhances the stability of the system. The bifurcation diagram in Fig. 22 demonstrates that the system maintains stable behavior within the range $0<f \leqslant 0.18$. This stability is corroborated by the PMs in Fig. 23, where a single red point represents the system’s periodic motion at $f=0.19$.

However, beyond $f>0.18$, the system transitions from a stable state to chaotic behavior. This transition is evident in Fig. 23, where the PMs show red points distributed randomly, indicating chaotic dynamics. These findings highlight the critical role of the control device in regulating the system’s dynamics and mitigating chaotic responses.

## Conclusion

The behavior of a dynamical system comprising two nonlinear damped harmonic springs attached to a piezoelectric EH device has been examined. To lessen any potentially dangerous vibrations, the NVF controller is utilized. The governing EOM are derived by applying LE, and the MSM is employed for analytic results up to third-order approximation. These findings have been mathematically confirmed using the RK- 4 technique and are supported by graphs. ME are acquired, and every external resonance case that has been obtained is assessed. The amplitude of the system is affected by different values of the damping coefficient, gains of the NVF controller, and excitation amplitude of an external force, as shown graphically. Furthermore, the stability of the system is demonstrated, and its stable and unstable fixed points are identified using the resonance response curves. It was determined that, because of the NVF controller represented by $( - G\,{\dot {x}_2})$ the amplitude of ${\dot {x}_2}(\tau )$ is decreased by $99.41\,\%$. This is useful as the controller’s primary objective is to lessen dynamic vibrations while simultaneously taking advantage of the voltage that the piezoelectric EH device produces. The influence of control devices on the chaotic dynamics of the system is analyzed, demonstrating their ability to maintain steady behavior before the system transitions to chaos. This is illustrated through bifurcation diagrams, PPs, and PMs. The dynamical system being studied is important because it may be applied to many different things, including medical sensors, consumer electronics (printers, speakers), industrial actuators, and microphones. Conversely, vibration control improves safety and performance by reducing undesired oscillations in mechanical systems. Passive, active, and hybrid control methods are utilized in buildings, vehicles, aerospace, and industrial equipment to minimize structural fatigue and enhance operational stability. So, the idea of converting energy harvesting vibrations into electrical energy, reduces battery dependence, while vibration control improves stability and efficiency. Their combination supports self-sufficiency systems, with future innovations leading to smarter and more sustainable engineering developments.

## Data Availability

The datasets used and/or analysed during the current study available from the corresponding author on reasonable request.

## Appendix I

$${H_1}=3{\lambda ^2}{\tilde {\alpha }_1}(8{(1+\lambda )^4}+3{B^2}{e^{2\,i{\tau _0}}}\lambda {\tilde {\alpha }_2}),$$

$${H_2}=E\,{e^{2\,i\,{\tau _0}}}(1 - 5\lambda - 5{\lambda ^2}+2{\lambda ^3}+{\lambda ^4}) - 8\lambda (B{e^{2\,i\,{\tau _0}}}+9\lambda +9){\tilde {\alpha }_2},$$

$${H_3}=1 - 5\lambda - 5{\lambda ^2}+2{\lambda ^3}+{\lambda ^4},$$

$${H_4}=4\lambda (3\lambda - 2)\tilde {\alpha }_{2}^{2}+3{\lambda ^3}{\tilde {\alpha }_1}(1+i+7{\tilde {\alpha }_2}),$$

$${H_5}=\frac{{3{E^2}\lambda \,{e^{ - \,i\,{\tau _0}}}}}{{8{{(1+\lambda )}^6}}}{(E\,{e^{2\,i\,{\tau _0}}}+\bar {E})^2},$$

$${H_6}=E\,{e^{2\,i\,{\tau _0}}}({\lambda ^3}+2{\lambda ^2}+2\lambda +1)+8\lambda {\tilde {\alpha }_2}(E{e^{2\,i\,{\tau _0}}}+3\bar {E}),$$

$${H_7}=E{e^{2\,i\,{\tau _0}}}(8 - 9\lambda ) - 24(\lambda - 1)\bar {E},$$

$${H_8}=6{\lambda ^2}{\tilde {\alpha }_1}(8{(1+\lambda )^4}+15{B^2}{e^{2\,i{\tau _0}}}\lambda {\tilde {\alpha }_2})+5E\,E\,{e^{2\,i\,{\tau _0}}}(1 - 5\lambda - 5{\lambda ^2}+2{\lambda ^3}+{\lambda ^4}),$$

$${H_9}=\frac{{\tilde {f}\,p\,{{\tilde {R}}_p}\omega \ell \tilde {\gamma }{e^{i\,p\,{\tau _0}}}}}{{128{{(1+\lambda )}^6}(1 - p)(1+i\,{{\tilde {c}}_p}{{\tilde {R}}_p}\omega p)}},$$

$${H_{10}}={E^2}\bar {E}\frac{{({{\tilde {\alpha }}_1}{\lambda ^3}+1)}}{{{{(1+\lambda )}^3}}}\frac{{{{\tilde {R}}_p}\omega \ell \tilde {\gamma }}}{{(1+3i\,{{\tilde {c}}_p}{{\tilde {R}}_p}\omega )}}[{(\frac{\lambda }{{1+\lambda }})^3}{\tilde {\alpha }_1}\,{B^2}\,{e^{3\,i\tau \,}}+\frac{{\,3{B^2}}}{{8{{(1+\lambda )}^3}}}\,{e^{3\,i\tau }},$$

$${H_{11}}=\frac{{{{\tilde {R}}_p}\omega \ell \tilde {\gamma }}}{{(1+3i\,{{\tilde {c}}_p}{{\tilde {R}}_p}\omega )}}\frac{{{{\tilde {c}}_p}{{\tilde {R}}_p}\omega }}{{(3{{\tilde {c}}_p}{{\tilde {R}}_p}\omega - i)}}\frac{{({{\tilde {\alpha }}_1}{\lambda ^3}+1)}}{{2{{(1+\lambda )}^3}}}9i{E^4}\bar {E}{e^{3i{\tau _0}}},$$

$$\begin{gathered} {H_{12}}=\frac{{15[ - 30{\alpha _1}{\alpha _2}{\lambda ^3}+\alpha _{1}^{2}{\lambda ^5}(16+\lambda )+\alpha _{2}^{2}(16\lambda +1)]}}{{256{{(\lambda +1)}^6}}}\end{gathered}$$ 

$${H_{13}}=\frac{{{R_p}^{2}{\omega ^2}{c_p}\mu \,\gamma }}{{2({R_p}^{2}{c_p}^{2}{\omega ^2}+1)}}, \hfill$$ 

$${H_{14}}=\frac{{{R_p}\omega \,\mu \,\gamma }}{{2({R_p}^{2}{c_p}^{2}{\omega ^2}+1)}}$$ 

$${d_1}=\frac{{9({\alpha _2}+{\lambda ^3}{\alpha _1})}}{{8{{(1+\lambda )}^3}}}a_{0}^{2},$$

$${d_2}=75a_{0}^{4}[\alpha _{1}^{2}{\lambda ^5}(\lambda +16)+\alpha _{2}^{2}(16\lambda +1) - 30{\alpha _1}{\alpha _2}{\lambda ^3}]/[256{(\lambda +1)^6}],$$

## Abbreviations

DAE: Differential-algebraic equations
EOM: Equations of motion
PM: Poincaré map
PP: Phase portrait
AS: Approximate solutions
NS: Numerical solutions
MSM: Multiple-scales method
NVF: Negative velocity feedback
EH: Energy harvesting
RK- 4: Fourth-order Runge–Kutta method
CVF: Cubic velocity feedback
m: The rigid body mass
$$V_E$$: Potential energy
T: Kinetic energy
F(t): External force
C: Damping coefficient
$R_p$: The resistive load associated with the piezoelectric circuit
$c_p$: Capacitance associated with the piezoelectric
γ: Piezoelectric circuit’s linear coupling coefficient
G: Negative controller with gains
V: The value of the load voltage$R_L$
$V_E, T$: The energies, both potential and kinetic
S: The point at which the springs are connected to each other
$L_{o1}+X_1(t)$: The position of the massless point S
$L_{o1}+L_{o2}+X_2(t)$: The position of the body
${L_{o1}}$,${L_{o2}}$: The nominal lengths of the springs
$P_{out}$: Output power generated by the piezoelectric circuit.
E: Energy released by the piezoelectric circuit.
